# Regaining Candidacy for Heart Transplantation after Robotic Assisted Laparoscopic Radical Prostatectomy in Left Ventricular Assist Device Patient

**DOI:** 10.1155/2012/716201

**Published:** 2012-10-09

**Authors:** Tariq A. Khemees, Ahmad Shabsigh

**Affiliations:** Department of Urology, The Ohio State University Wexner Medical Center and James Cancer Hospital, Columbus, OH 43212, USA

## Abstract

Several factors may highlight the relevance of prostate cancer to the pre-heart-transplant population. First, the expansion in candidate selection criteria led to increased number of men over the age of fifty to be considered for heart transplantation. With the introduction of left ventricular assist device (LVAD) therapy, waiting-list mortality has dramatically declined over the past decade. Additionally, transplant candidates are diligently screened for preexisting neoplasm while on the waiting list. Taken together, screening-detected prostate cancer may increasingly be diagnosed in patients on the waiting list. If discovered, it will pose unique challenge to clinicians as to date there has been no universally accepted management guideline. We report a case of LVAD-treated heart transplant candidate diagnosed with prostate cancer while on the waiting list. Patient screening demonstrated PSA elevation which prompted prostate biopsy. Low-risk clinically localized prostate cancer was confirmed and led to removal of patient from transplant list. When counseled regarding management of his cancer, the patient elected to undergo radical prostatectomy in a hope to regain candidacy for heart transplantation. Despite being of high surgical risk, multidisciplinary team approach led to successful management of prostate cancer and the patient eventually received heart transplant one year following prostatectomy.

## 1. Introduction

Despite an estimate of 20,000 to 30,000 heart transplantation (HTx) candidates, only 2,322 HTx procedures were performed during 2011 in the United States [1, 2]. Organ shortage is the main cause for this disparity. While on the waiting list, transplant candidates may not survive until a matching donor is available, or may suffer potentially reversible transplant contraindications making them excellent candidates for mechanical circulatory support with left ventricular assist devices (LVADs) [3]. The expanded application of LVAD therapy over the past decade has led to a significant improvement in waiting-list mortality [4].

To optimize transplantation outcomes and improve patient morbidity and mortality, thorough medical evaluation of the recipients is strictly enforced by transplant centers [5]. With active malignancy being a contraindication to transplantation, screening programs for preexisting neoplasm are applied while on the waiting list [5, 6]. These programs are usually extrapolated or modified from the general population guidelines.

In the USA, up to 45% of HTx recipients are men over the age of fifty [2] who may get diligent screening for prostate cancer (PC) per the selection guidelines [5]; however, the lack of evidence of a proven benefit of PC screening and the controversy of screening men with limited life expectancy have led the United States Preventive Services Task Force (USPSTF) to recommend against prostate-specific-antigen-(PSA-) based PC screening programs for US men [7]. Despite these new recommendations, however, it is the standard for most candidates to be screened according to the conventional protocol, that is, yearly PSA and digital rectal examination in men over the age of fifty. Considering the natural history of PC, applying such screening might result in an increased identification of small, indolent, and clinically localized PC that would never have caused symptoms and/or resulted in a significant 10-year cancer-specific mortality in patients awaiting transplantation [8, 9]. Furthermore, it is not uncommon for screened candidates to be removed from the waiting list due to the detection of PC [6]. This would ultimately result in unnecessary delay of the transplant procedure as the cause-specific mortality for such cancers is decimal when compared to the mortality risk subsequent to HTx.

Multiple strategies are available to treat localized PC including active surveillance, radiotherapy, and surgery [10]. For a patient from the general population, the main deciding factors in selecting treatment modality are cancer control and the potential side effects of the therapeutic procedure with subsequent effects on his quality of life [11]. This decision making process might not be applicable to the transplant candidate diagnosed with screening-detected PC while on the waiting list. Currently, there are no established guidelines addressing management of the disease in pretransplant population. While the 2006 International Society for Heart and Lung Transplantation Guidelines recommend considering HTx in a patient with preceding low-risk PC [5], some transplant centers require treatment of the disease and documentation of cancer-free interval (i.e., no clinical or biochemical recurrence) before listing the patient as a candidate. The waiting interval before reconsideration for HTx is usually related to oncologic outcomes and estimated risk for PC recurrence. This will pose a special therapeutic dilemma as patient's decisions might be driven by selecting a modality that would offer the shortest waiting for enlistment as a transplant candidate. In this paper we present a case of a patient who was taken off the waiting list due to his clinical diagnosis of biopsy-proven low-risk clinically localized PC.

## 2. Materials and Methods 

### 2.1. Case Presentation

 A 60-year-old African American male presents with NYHA class III ischemic heart failure managed in 2011 with HeartMate II LVAD as a bridge to HTx and implantable cardioverter defibrillator (ICD). Patient screening revealed elevated PSA of 8.38 ng/dL and digital rectal examination estimated prostate volume at 90 g with a suspicious nodule in the right lobe. Transrectal ultrasound-guided prostate biopsy (TRUS) revealed Gleason score 3 + 3 = 6 adenocarcinoma in one out of fourteen biopsy cores with <15% of that core involved with cancer. After this new diagnosis, the transplant team decided to take the patient off the waiting list pending treatment of his PC.

Medical history includes atrial fibrillation, hypertension, hyperlipidemia, asthma, type 2 diabetes mellitus, deep venous thrombosis, dyspepsia, and erectile dysfunction. Medication list includes metoprolol, atorvastatin, fluticasone, sitagliptin, warfarin, aspirin, finasteride, tamsulosin, and omeprazole. Surgical history includes total colectomy with ileostomy in 2009 due to ischemic colitis. The patient had 17-year smoking history of half pack per day with no alcohol or illicit drug intake. He is married and has 9 children.

Therapeutic options for localized PC were discussed with the patient and with the transplant team. The patient was educated regarding risks and benefits for each option. He was informed about the greater perioperative risk due to his comorbidities. He elected to proceed with surgical excision in a hope to regain candidacy for HTx.

### 2.2. Perioperative Management

Giving the patient's challenging cardiac risk, a multidisciplinary case conference was held with a panel of transplant cardiothoracic surgeon, transplant cardiologist, cardiac anesthesiologist, oncologist, and urologist. The consensus from the panel was that if the patient received any treatment but surgical resection for PC, he will need to wait for at least two years before being considered for HTx and surgery may allow for quickest return to the waiting list. The hemodynamic effect of Trendelenburg positioning was discussed with the perfusionist and anesthesiology teams. Monitoring during surgery required establishing an arterial pressure line, central venous line, pulmonary artery catheter, transesophageal echocardiography (TEE), esophageal temperature probe, serial blood gas analyses, EKG, and pulse oximetry. Results from these tests along with LVAD console parameters were used as surrogate to guide and maintain safe cardiac output throughout surgery. Cardiac defibrillator pads were placed and the ICD was turned off preoperatively.

As the presence of LVAD pump requires continued anticoagulation during surgery, we believed a minimally invasive approach would help lower surgical blood loss and reduce operative time. Anticoagulation management included withholding warfarin therapy two days prior to surgery and switching to 5000 I.U. subcutaneous heparin six hourly while continuing his aspirin therapy. The patient's international normalized ratio (INR) was 2 at time of surgery. 

### 2.3. Surgical Procedure

The patient was placed in dorsal lithotomy with steep Trendelenburg position. Sequential compression devices were applied to both legs. A prior laparatomy scar necessitated access to the peritoneal cavity via Veress needle incision at the left lower quadrant. 15 mm Hg pneumoperitoneum was obtained with CO2 gas. Extensive adhesions were encountered upon entry into peri-umbilical and pelvic areas but were able to be managed laparoscopically to allow safe placement of other ports. The robot, da Vinci S system (Intuitive Surgical, Inc. Sunnyvale, CA, USA), was docked and radical prostatectomy was done in standard fashion. Of note, we encountered some difficulty in maintaining hemostasis during dissection due to continued blood oozing from surgical planes adding to the operative time. Furthermore, a large median lobe necessitated careful bladder neck dissection with a safe distance from both ureteric orifices. A watertight vesicoureteral anastomosis was achieved over two-way 20 Fr. Foley catheter. Given his baseline erectile dysfunction, a non-nerve-sparing approach was used in an attempt to lower operative time and blood loss. Due to the low-risk nature of PC and adhesions encountered on the pelvis side walls, no pelvic lymph node dissection was performed. Total operative time was 400 minutes and estimated blood loss was 300 mL.

The postoperative course was uncomplicated and the patient discharged home on postoperative day (POD) 8 after reinstating oral anticoagulant medications. Cystogram done on POD 11 demonstrated no urinary extravasation. Pathology report indicated a prostate weight of 115 g with stage pT2c, Gleason score 3 + 3 = 6 with negative surgical margins. One month after surgery, the PSA nadir was 0.01 ng/mL. A year after surgery, the patient was fully continent (using zero pads per day) and his PSA continues to be undetectable. His candidacy for HTx was reinstated. Ultimately, the patient successfully received his orthotopic HTx at our center.

## 3. Discussion

To date, there have been no universally accepted standards of care for localized PC in pretransplant population. The series from Israel Penn International Transplant Tumor Registry represent the largest available data on patients treated for PC prior to solid organ transplantation [12]. Ten patients in this study received HTx subsequent to PC treatment. High rates of disease progression and mortality were reported after transplantation and can potentially justify screening for PC before transplant procedures. However, multiple aspects of concerns can be raised when examining these data. First, the study is limited by the lack of reporting pathology and PSA information which are essential for patient risk stratification. These data represent the cornerstone in predicting risk of PC recurrence and may potentially explain the overall higher rates of disease progression and mortality when compared to other more detailed reports in posttransplant patients [13, 14]. Lastly, there was heterogeneity in reporting therapeutic modalities with lack of details on their respective indications.

Available evidence supports safety and feasibility of active surveillance for men in the general population diagnosed with localized PC with a low probability of cancer-related death during the first 10 years after diagnosis [15]. When considering the 70% probability for the five-year survival in men over the age of fifty who received HTx [2], one might argue for a meaningful and significant survival benefit of active surveillance if offered to treat screen-detected PC while on the waiting list. Nonetheless, the conceivable risk for this approach is that transplant patients are kept on immunosuppressant after the procedure which carries the risk of promoting cancer progression [16]. Until further studies detailing how immunosuppression impacts the natural history of PC, however, the significance of this risk remains unclear.

Radiation therapy (both external beam and brachytherapy) is potentially curative treatment for localized PC. However, several aspects limit its applicability in this setting. First, therapeutic response of PC to radiotherapy is rather slow and histological regression of these slowly growing cancers may take up to three years to be completely achieved [17]. Second, patients treated with radiation may maintain detectable PSA levels years after treatment making it difficult to monitor for recurrence following therapy [18]. Additionally, brachytherapy is generally reserved for PC in gland of <60 g without evidence of extraprostatic disease extension on preoperative imaging [11].

Radical prostatectomy before HTx would be the most logical option to treat PC in the pre-transplant patients who need therapy for multiple reasons. First, specimen examination after surgical resection will provide accurate pathological staging. This information will facilitate risk stratification for tumor progression. Second, rapid PSA nadir after surgery and simpler PSA kinetic afterward will enable monitoring for biochemical failure and disease progression. Furthermore, delaying surgery until after HTx carries the risk of impaired wound healing due to the effect of immunosuppression needed after transplantation. In our patient, the large prostate volume precluded the applicability of both brachytherapy and external beam radiotherapy, unless several months of androgen deprivation therapy were utilized to shrink his gland to a suitable volume for the procedures. In addition, low-risk nature of PC confirmed by examining surgical specimen estimated PC recurrence risk within next 10 years to be less than 3% [19].

Our results are interesting in multiple aspects. To our knowledge, this is the first report of a patient treated with two new technologies: mechanical circulatory support with LVAD and minimally invasive RARP. Intensive monitoring during surgery helped prompt correction of any derangement in cardiac output while the robotic approach minimized operative time and blood loss. Also, successful management of PC resulted in the patient receiving his HTx in a timely fashion with minimum delay. Lastly, with the paucity of data addressing treatment of PC before solid organ transplantation, our result will add to the available evidence guiding clinicians to succeed in similar clinical scenarios.

## 4. Conclusion

Our results suggest that, in the setting of screen-detected PC in a pre-transplant candidate, radical prostatectomy can be safely achieved even for high-risk LVAD-treated patients. Pathological data provided from surgical specimen helped in accurate assessment of disease progression and rapid PSA drop following surgery provided sensitive tool to monitor for PC recurrence. Unnecessary delay or denial of HTx procedure was avoided.

## Abbreviations

HTx: Heart transplantation
ICD: Implantable cardioverter defibrillator
LVADs: Left ventricular assist devices
PC: Prostate cancer
PSA: Prostate-specific antigen
RARP: Robotic assisted radical prostatectomy.
